# Psychometric evaluation of the Russian version of the flourishing scale in a sample of older adults living in Siberia

**DOI:** 10.1186/s12955-019-1100-6

**Published:** 2019-02-11

**Authors:** Daniele Didino, Ekaterina A. Taran, Galina A. Barysheva, Fabio Casati

**Affiliations:** 1 Department of Psychology, Humboldt-Universität zu Berlin, Rudower, Chaussee 18, 12489 Berlin, Germany; 2 School of Engineering Entrepreneurship, Tomsk Polytechnic University, Tomsk, Russia; 3 Department of Information Engineering and Computer Science, University of Trento, Trento, Italy

**Keywords:** Flourishing scale, Psychological well-being, Older adults, Russian version, Psychometric assessment

## Abstract

**Background:**

The development of measures of psychological functioning has received less attention in Russia compared to other countries. Moreover, despite the growing interest in the psychological well-being of older adults, there is a lack of research on this segment of the population in Siberia. Therefore, in this study we aimed to evaluate the psychometric properties of the Russian version of the Flourishing Scale (FS) and to measure psychological functioning in a sample of older adults living in Siberia.

**Methods:**

The FS was translated and adapted to Russian language and culture using the back-translation procedure. The Russian version was administered to 851 older adults (aged from 65 to 93 years, mean = 75.3; female = 510). A principal axis factor analysis (PFA) and a confirmatory factor analysis (CFA) were used to assess the structural validity. Cronbach’s alpha coefficients (internal consistency) and item-total correlations were also analysed. To test the convergent validity, the FS was compared with other scales assessing mental well-being.

**Results:**

The PFA and the CFA showed that the FS had good psychometric properties. A one-factor structure was a good model of fit, even if some items had a low loading (ranging between 0.39 and 0.80). The Cronbach’s alpha value was 0.82 and the Cronbach’s alpha values if an item were deleted ranged from 0.78 to 0.81. The item-total correlation coefficients ranged from 0.54 to 0.76. The FS also showed good convergent and divergent validity with other scales (correlation coefficients ranging from 0.39 to 0.54). The mean flourishing score (40.9) reported by the older adults in our sample is lower than that reported in previous studies.

**Conclusions:**

The Russian version of the FS seems to have good psychometric properties and to be a valid and reliable instrument to evaluate flourishing among Russian older adults. This study provides the first evaluation of an instrument that Russian researchers and policymakers can use to better understand the determinants of successful ageing in this society. Future studies should further assess the validity and reliability of the FS and should generalize these results to different groups (e.g., adolescent or workers).

**Electronic supplementary material:**

The online version of this article (10.1186/s12955-019-1100-6) contains supplementary material, which is available to authorized users.

## Background

Understanding the factors that influence the quality of life of older adults is a fundamental step in developing policy decisions that allow us to maintain physical, mental, and social well-being as we age. To investigate the predictors of well-being, it is important to develop valid and reliable instruments aimed at measuring the different components of this construct. The adaptation of these instruments to different cultures is also important to understand how the factors that influence well-being change for different cultures. In this study we evaluated the psychometric properties of the Russian version of the Flourishing Scale (FS, [1]) in a sample of older adults living in Tomsk (Siberian Federal District, Russia).

Although a substantial amount of research has been conducted to investigate well-being of older adults [2], little attention has been given to older adults living in the Siberia [3]. Well-being is influenced by socio-economic and cultural factors [4] and in this regard the peculiarity of the socio-cultural context in which Siberian older adults live provides an interesting setting to evaluate the cross-cultural validity of the FS. In fact, Siberian older adults are part of a multi-ethnic and multicultural society, which has been influenced by both Western and Eastern cultures [3]. Moreover, Russian older adults have been subjected to the effects of the extensive social, political and economic changes occurred in the decades after the breakup of the USSR [5–7]. This transition had long-term negative outcomes on many aspects of the society (e.g., material hardship, lack of social cohesion, lower perceived control over one’s own life, and poor psychophysical health) and increased socioeconomic inequalities, poverty, and unemployment [5].

Well-being is a complex construct that describes the individual’s quality of life by considering different cognitive, emotional, and psychological aspects [4, 8–12]. Current research conceptualizes this construct as a multidimensional concept and two major views of well-being emerged based on two historical approaches [4]. The *hedonic* view considers pleasure, happiness, and pain avoidance as central aspects to define subjective well-being [4]. Subjective well-being has thus been operationalized as the synthesis of three components [13]: life satisfaction (i.e., the person’s cognitive judgement of his/her life), the presence of positive feeling (i.e., positive affect), and the absence of negative feeling (i.e., negative affect). The *eudaimonic* view focuses on human potential and includes concepts as personal growth, self-realization, and purpose or meaning in life [4, 11, 12]. Eudaimonic research considers positive psychological functioning and prosperity as a central element of psychological and social well-being [10, 11, 14, 15]. Although hedonic and eudaimonic are two distinct dimensions of well-being [11], the scales developed to measure these constructs tend to correlate. For example, the FS (which measures psychological well-being) and the Satisfaction With Life Scale (SWLS, which measures subjective well-being [16]) positively correlate [1].

Although some instruments to measure subjective well-being are available in Russian language (e.g., SWLS, [16]), much less attention has been devoted to psychological well-being. In fact, to our knowledge, no measure of eudaimonic well-being has been validated in a Russian sample. In this paper, we present the Russian version of the FS [1], a short scale developed to measure psychological well-being in term of psychological functioning. A high level of flourishing is related to a large set of individual and social positive outcomes, such as better health, being more productive, and higher life expectancy [9, 17, 18]. Therefore, it is important to have valid and reliable measures of flourishing available to both researchers and policymakers.

The FS has been designed to briefly and easily assess psychological well-being (i.e., eudaimonic well-being) [1]. This scale includes eight positively phrased items (the English and Russian versions of the items are reported in Appendix I), which measure a wide set of aspects of respondent’s life. It has been already validated and used in different languages such as English [1, 19–22] (for the English version assessed in India see [23]), Chinese languages [24–27], Dutch [28], French [29], Iranian [30], Italian [31], Japanese [32], Malaysian [33], Portuguese [34], and Spanish [35–38]. However, further studies are required to confirm the invariance of its psychometric properties across different cultures.

Previous studies have shown that the FS has good psychometric properties, a high internal consistency and a high convergence with other well-being scales [1, 20–29, 31–36, 38]. Principal axis factor analyses performed in these studies revealed one strong factor with an eigenvalue ranging from 3.77 to 5.96, with the percentage of variance explained ranging from 42.3 to 74.46, and with factor loadings ranging from 0.42 to 0.91 [1, 20, 23, 26, 32–35, 38]. Confirmatory factor analyses showed factor loadings ranging from 0.32 to 0.91 [20–28, 31–35, 38]. Moreover, the FS also had high values of Cronbach’s alphas, which ranged from 0.78 to 0.95 [1, 20–36]. Finally, the scale showed high convergent validity with other well-being measures (e.g., SWLS [16]) and high divergent validity with measures of ill-being (e.g., Centre for Epidemiological Studies – Depression Scale, CES-D, [39]).

In most of the studies that administered the FS, the sample consisted of adults or young adults. In our knowledge, only two studies specifically assessed the psychometric properties of the FS in an older adult sample (i.e., individuals aged 65 or older). Hone and colleagues [20] analysed data from a New Zealand national survey with participants’ age ranging from 18 to 111. The mean score of the FS for older adults in the range 60–69 years was 45.19 (SD = 8.02), in the range 70–79 years was 46.51 (SD = 6.60), and in the range 80 or older was 43.22 (SD = 8.78). Considering that the score ranges from 8 to 56, older adults self-reported a high level of psychological well-being, suggesting successful functioning in the socio-psychological aspects of their lives. Momtaz and colleagues [33] interviewed a large sample of Malaysians older adult and categorized them into different well-being levels using a combined measure of the FS and the Geriatric Depression Scale [40]. Approximately 50% of the respondents were classified as fully flourishing (i.e., flourishing score above 47 and no signs of depression). The results of a regression analysis showed that well-being was significantly associated with gender, employment status, educational level, having children, and health. However, the dependent variable was a combination of two scales and no results were reported for the FS separately. The level of psychological well-being in older adults is probably related to the local socio-economic and cultural situation and thus different countries could show different patterns. Russian older adults have been subjected to the effects of the extensive social, political and economic changes occurred in the last decades after the breakup of the USSR [5–7]. Indeed, the World Happiness Report shows that people living in former Soviet Union countries experience a dramatic drop in life satisfaction as they age, a trend that continues in the years after retirement, unlike what happens in many other countries [41]. In a previous study, Didino and colleagues [7] measured subjective well-being in a sample of older adults living in Tomsk Region (respondents analysed in that previous study and in the current manuscript are from two different samples). The mean values of life satisfaction and happiness reported by Siberian older adults were relatively low compared to those reported in European countries [7]. It is plausible to assume that psychological well-being has also been negatively influenced by the long-term outcomes of the breakup of the USSR. However, no study measured psychological well-being in Siberian older adults. The current study addresses this issues by providing the first validation of the FS and reporting the first assessment of psychological well-being in a sample of Siberian older adults.

This study had two aims. First, to assess the validity and reliability of the Russian version of the FS. This is a novel measure which requires further studies to fully evaluate its psychometric properties across different countries and groups. Moreover, in Russia the development of measures of psychological functioning has received less attention and the adaptation of the FS can be useful both to investigate this aspect of well-being in the Russian population and to compare the results with those of other countries. The second objective was to investigate flourishing in Siberian older adults. Despite the growing interest in this segment of the population, there is a lack of research on Siberian older adults compared to Western countries. Given the particular socio-economic situation faced by Siberian older adults during their life, the level of flourishing in our sample might be lower compare to those of older adults living in other countries.

## Material and methods

### Respondents and procedure

A sample of 851 Russian older adults (aged from 65 to 93 years, M = 75.36, SD = 6.94) living in Tomsk (Siberian Federal District, Russia) were interviewed. The eligible criteria were being 65 years old or older and of Russian mother tongue. Demographic characteristics, self-reported health status, and satisfaction with standard of living are shown in Table 1. Consistently with the different life expectancy in the two sexes (women = 76.3 years, men = 64.7 years [42]), our sample includes more women (women = 510, men = 341).

**Table 1:** Demographic characteristics of the participants (*N* = 851) and mean, standard deviation and range of the flourishing score

Characteristics	Mean (SD) or N (%)	Flourishing score		
		Mean (SD)	Range	Statistics
Age	75.36 (6.94)			*β* = − 0.11^∗^
Female	76.16 (6.85)			*β* = − 0.13^∗^
Male	74.16 (6.90)			*β* = − 0.09
Sex				
Female	510 (59.93)	41.07 (6.83)	17–56	*χ*^2^(1) = 0.64
Male	341 (40.07)	40.68 (7.40)	21–56	
Marital status				
Divorced	66 (7.76)	41.32 (7.21)	27–54	*χ*^2^(3) = 9.28^∗^
Married/Partner	335 (39.37)	41.56 (7.48)	21–56	
Single	17 (2.00)	37.88 (6.91)	25–52	
Widowed	433 (50.88)	40.47 (6.66)	17–56	
Education				
High (university)	503 (59.11)	42.04 (6.58)	21–56	*χ*^2^(1) = 31.87^∗^
Low (less than university)	348 (40.89)	39.28 (7.42)	17–56	
Health				
Very bad (1)	68 (7.99)	35.50 (7.04)	24–51	*χ*^2^(4) = 107.01^∗^
Bad (2)	270 (31.73)	38.81 (6.75)	17–55	
Fair (3)	456 (53.58)	42.36 (6.48)	19–56	
Good (4)	54 (6.35)	46.04 (6.16)	32–56	
Very good (5)	3 (0.35)	40.67 (8.33)	34–50	
Satisfied Living Standard				
Satisfied	380 (44.65)	42.31 (6.94)	17–56	*χ*^2^(1) = 24.87^∗^
Dissatisfied	471 (55.35)	39.79 (6.97)	21–55	
Living arrangement				
Living alone	337 (39.60)	40.39 (6.59)	19–56	*χ*^2^(2) = 6.68^∗^
Living with relatives	507 (59.58)	41.31 (7.35)	17–56	
Living with non-relatives	7 (0.82)	37.43 (6.16)	29–47	

The relationship between flourishing score and age was measured with a linear model (flourishing score as outcome and age as predictor), for the overall sample and for the two sexes separately. The relationship between FS and the categorical variables was examined using Kruskal-Wallis rank sum tests. * *p* < 0.05

The current cross-sectional study was conducted in Tomsk. This city has a population of about half a million people and about 10 % (approximately 52,000 people, of whom approximately 70% are female) is 65 years or older. Door-to-door interviews were administrated within randomly selected neighbourhoods and streets of Tomsk. Interviewers were instructed to attempt to contact a family unit every 2 doors (in building with less than 25 flats), every 5 doors (26–50 flats), or every 10 doors (more than 50 flats), and to ask whether one of the people living in the residence was aged 65 or over and whether this person agreed to participate in the study. However, in case of a negative response (e.g., no member of the family unit was 65 years or older, or the older adult did not agree to participate), the interviewers were instructed to attempt to contact the family unit living in the next flat (i.e., the successive door). Face-to-face interviews were administered in respondent’s homes and in each family unit only one older adult was interviewed. All interviews were conducted in Russian by native speakers. Each interview lasted 40–60 min. Interviews were administered between May and June 2016 by an organization with experience in survey research - Research Center “Context” [Исследовательский центр “Контекст”]. Verbal informed consent was obtained from all respondents prior to the interview. Respondents were informed that their participation was voluntary and that they could withdraw from the interview at any time.

### Translation and adaptation

The translation and adaptation of the English version of the FS to Russian language and culture was performed using the back-translation procedure [43]. The translation was performed by two Russian mother-tongue translators (first translation) and by two English mother-tongue translators (back-translation). We ensured to reach equivalence between the original English and final Russian versions. After this procedure, a pre-final version of the FS was tested with 10 older adults. Suggested changes were discussed in detail by our research group with the translators that took part in the adaptation process, resulting in the final version of the scale. This final version was tested again on 20 older adults and no comprehension problems were found. These 20 respondents interviewed with the final version were included in the sample. The final Russian version of the FS is reported in Appendix I.

### Measures

#### Flourishing scale (FS)

The FS is an 8-item measure that assesses important aspects of psychological functioning. Each item uses a 7-point Likert scale ranging from 1 (“*strongly disagree*”) to 7 (“*strongly agree*”), see Appendix I. The total flourishing score is given by the sum of the scores of the single items and ranges from 8 to 56. Higher scores correspond to higher social-psychological flourishing. All items are positively phrased.

#### Satisfaction with life scale (SWLS)

The SWLS [16] is a 5-item measure aimed at assessing the overall satisfaction with one’s life. Each item uses a 7-point Likert scale from 1 (“*strongly disagree*”) to 7 (“*strongly agree*”). The total score is given by the sum of the scores of the single items and ranges from 5 (extremely dissatisfied) to 35 (extremely satisfied). The SWLS has been translated and adapted to Russian language by Tucker and colleagues [44]. In the current study, the Cronbach’s alpha of the SWLS was 0.79.

#### Center for Epidemiologic Studies–Depression Scale (CES-D)

The CES-D 8 [45] is 8-item measure developed to assess the frequency of depressive symptoms over the past week. Each item uses a 4-point Likert scale from 0 (“*none or almost none of the time*”) to 3 (“*all or almost all of the time*”). The total score is given by the sum of the scores of the single items and ranges from 0 to 24. Higher scores indicate a higher frequency of depressive symptoms. The Russian version of the CES-D 8 scale can be found in the European Social Survey [46] (see also [47]). In the current study, the Cronbach’s alpha of the CES-D 8 was 0.80.

#### Life satisfaction (LS) and happiness (HS)

LS has been assessed with the single item: “*All things considered, how satisfied are you with your life as a whole these days? Use a 0 to 10 scale, where 0 is dissatisfied and 10 is satisfied.*” Higher scores indicate higher perceived life satisfaction. HS has also been assessed with the single item: “*Taking all things together, how happy would you say you are?*” Respondents could use a scale from 0 (extremely unhappy) to 10 (extremely happy). Higher scores indicate higher level of perceived happiness. The Russian translation of both items can be found in the European Social Survey [46].

#### Self-rated health, living conditions, and demographic variables

Perceived health was measured with the question: “*How is your health in general? Would you say it is ...?*” Respondent could use the following scale: 1 = “*very bad*”; 2 = “*bad*”; 3 = “*fair*”; 4 = “*good*”; 5 = “*Very good*”. Satisfaction with standard of living was measured with the question: “*Are you satisfied or dissatisfied with your standard of living all the things you can buy and do?*” Respondent could choose between “*Satisfied*” or “*dissatisfied*”. Both questions have been selected from the Russian version of the Gallup World Poll [48]. Demographic variables were: Age, sex, marital status (“*divorced*”, “*married/living with a partner*”, “*single*”, “*widowed*”), educational level (low – “*less than university*”, or high – “*university or more*”), and living arrangement (“*living alone*”, “*living with relatives*”, “*living with non-relatives*”).

### Statistical analysis

Data were analysed using the R-project software [49], RStudio software [50], and the R-package lavaan [51] (for a template of the R script see “Additional file 1.R”). A principal axis factor analysis and a confirmatory factor analysis were performed to assess a one-factor model, based on previous studies [1, 20, 21, 25, 26, 29, 32–34]. In the confirmatory factor analysis, standard fit indices were used to test the model: Chi-square value, normed Chi-square value, the root mean square error of approximation (RMSEA), the standardized root mean square residual (SRMR), the goodness-of-fit index (GFI), the comparative fit index (CFI). The acceptable cutoff values for the indices are: normed chi-squared in the range 2.0–5.0, RMSEA in the range 0.06–0.08, SRMR < 0.08, GFI > 0.9, CFI > 0.9 [52, 53].

Internal consistency was evaluated using the Cronbach’s alpha. An alpha value less than 0.7 indicates poor internal consistency, a value between 0.7 and 0.9 indicates an acceptable internal consistency, and a value greater than 0.9 indicates an excellent internal consistency [54]. Cronbach’s alpha was chosen for comparison with reliability reported in previous studies. The item-total correlations were calculated with the R-package psych [55]. Convergent and discriminant validity was analysed assessing the correlation (Spearman’s *ρ*) between the FS and the SWLS, the HS, the LS, and the CES-D 8. All data analysed in this manuscript are presented in the supplementary file “Additional file 2.csv”.

## Results

### Descriptive analysis and internal consistency

**Fig. 1:**
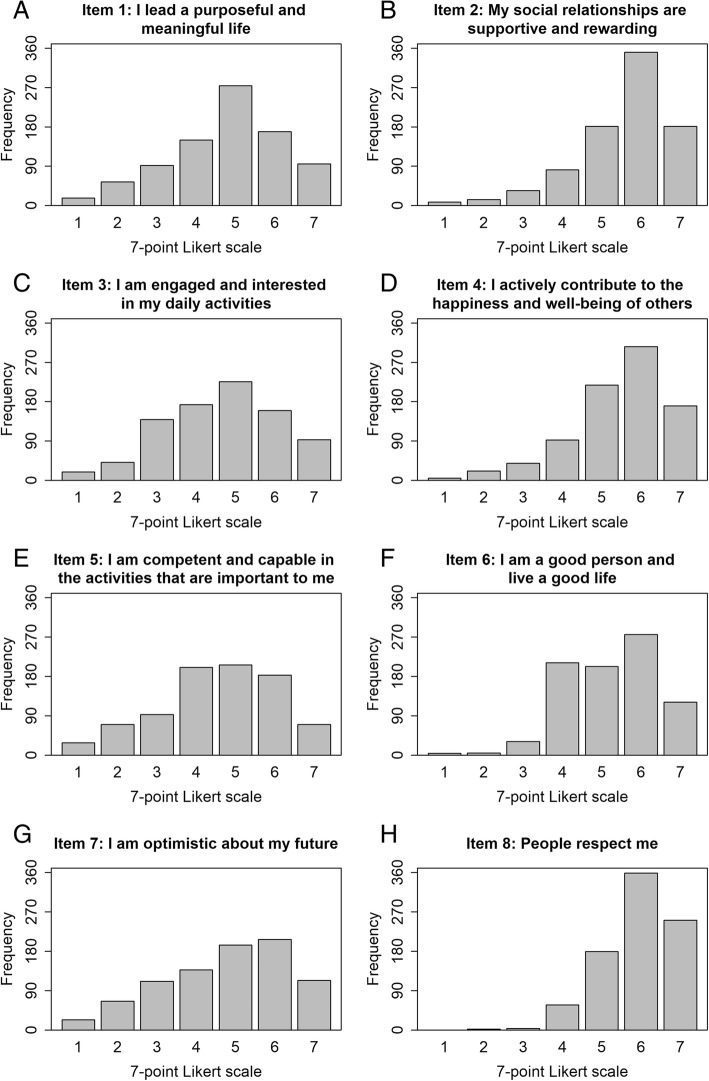
Distribution of the eight items (panels **a** to **h**) of the FS. The items are reported above the corresponding histogram

**Fig. 2:**
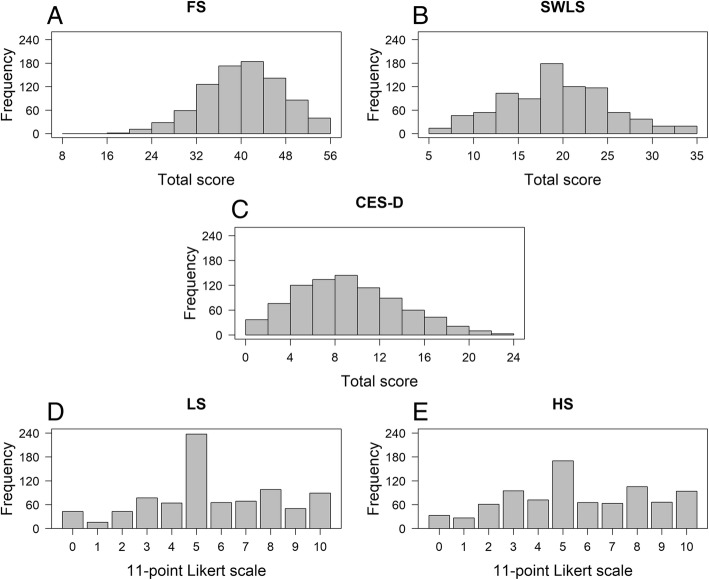
Distribution of the total score of the scales. **a**: Flourishing Scale (FS); **b**: Satisfaction With Life Scale (SWLS); **c**: Center for Epidemiologic Studies–Depression Scale (CES-D); **d**: Life Satisfaction (LS); **e**: Happiness (HS)

Table 1 reports the flourishing score for the demographic variables, self-reported health, and satisfaction with standard of living. For the overall sample and for female respondents, flourishing decreased with age. Moreover, except sex, all the other demographic characteristics (marital status, educational level, perceived health, satisfaction with standard of living, living arrangement) influenced the flourishing score. The response distribution of the items of the FS (Fig. 1) and the distribution of the total scores of the scales (Fig. 2) are reported in Appendix II. Table 2 shows the statistics of the scales. The total flourishing score ranged from 17 to 56 (mean = 40.9, SD = 7.1). For the FS, the mean values for the single items ranged from 4.5 to 5.9 (Table 3). This suggests that respondents had a positive perception of their flourishing, even if lower than that reported by older adults in previous study [20]. To test this, the mean flourishing scores in our sample and those reported in Table 1 of Hone and colleagues’ study [20] were compared with a Welch’s t-test. To compare the two studies, the mean flourishing score in our sample has been calculated for each age groups reported in Hone and colleagues’ study (Table 4). In each age group, the mean flourishing score reported by our sample was significantly lower than that reported in Hone and colleagues’ study (all *p*s < 0.05, see Table 4). Cronbach’s alpha value for the FS was 0.82, which is within the range of values (i.e., from 0.78 to 0.95) reported in previous studies [1, 20–36]. The Cronbach’s alpha values if an item is deleted ranged from 0.78 to 0.81 (Table 3), which indicates an acceptable internal consistency. Finally, the item-total correlation coefficients ranged from 0.54 to 0.76 (Table 3).

**Table 2:** Correlation (Spearman’s *ρ*) between the scales (all *p*s < 0.001). The two rightmost columns show the statistics of the scales

	FS	SWLS	CES-D 8	HS	LS	Mean (SD)	Range
FS	–					40.9 (7.1)	17–56
SWLS	0.54	–				19.5 (6.0)	5–35
CES-D 8	−0.49	−0.43	–			9.7 (4.7)	0–24
HS	0.50	0.57	−0.46	–		5.6 (2.8)	0–10
LS	0.39	0.54	− 0.38	0.67	–	5.6 (2.7)	0–10

**Table 3:** Statistics and internal reliability of the items: Mean values (standard deviations), factor loadings (confirmatory factor analysis), item-total correlations, and internal consistency (Cronbach’s alphas if item deleted)

Item	Mean (SD)	Loading	Item-total correlation	α if item deleted
Item 1: I lead a purposeful and meaningful life	4.8 (1.4)	0.76	0.75	0.78
Item 2: My social relationships are supportive and rewarding	5.6 (1.2)	0.39	0.54	0.81
Item 3: I am engaged and interested in my daily activities	4.6 (1.5)	0.80	0.76	0.78
Item 4: I actively contribute to the happiness and well-being of others	5.5 (1.2)	0.53	0.66	0.79
Item 5: I am competent and capable in the activities that are important to me	4.5 (1.5)	0.62	0.66	0.80
Item 6: I am a good person and live a good life	5.3 (1.2)	0.42	0.60	0.80
Item 7: I am optimistic about my future	4.7 (1.6)	0.58	0.71	0.79
Item 8: People respect me	5.9 (0.9)	0.44	0.62	0.80

**Table 4:** Welch’s t-test comparing the flourishing scores in our sample and those reported in Hone and colleagues’ study [20]

Age	Our sample		Hone et al.’s sample		*t*	df	*p*-value
	Mean (SD)	N	Mean (SD)	N			
60–69 years	41.29 (6.81)	235	45.19 (8.02)	1344	7.88	357.59	< 0.001
70–79 years	41.35 (7.01)	378	46.51 (6.60)	492	11.04	785.6	< 0.001
80 years and over	39.85 (7.31)	238	43.22 (8.78)	54	2.62	70.59	< 0.05

### Factor validity

A principal axis factor analysis of the FS revealed one strong factor with an eigenvalue of 3.56 that accounted for the 37% of the total variance. A second factor was slightly greater than 1 (1.03) but a visual inspection of the scree plot [56] revealed a clear break between the first and the second component. The factor loadings ranged from 0.46 to 0.71. These results indicate that only one factor characterised the FS.

A confirmatory factor analysis was conducted to test whether a single-factor structure fits the data of the FS. In the initial baseline model (Model 1), we did not allow error correlations between items. Similar to previous studies [20–22, 27, 33], this model showed a poor fit (see Table 5). In fact, the normed chi-squared, the RMSEA, and the CFI failed to reach the acceptable threshold values. Therefore, to improve the goodness of fit of the model, we allowed error correlations by using the standard modification indices procedure [57, 58]. The error correlations were sequentially added to the models (see Models 2–7 in Table 5). The final model had a better fitting and all the indices had satisfactory values within the acceptable range, showing that a one-factor model was a good model of fit and was able to describe the factor structure of the scale. The standardized factor loadings for the FS are reported in Table 3 and ranged from 0.39 to 0.80, which are all statistically significant (*p* < 0.001). These results confirmed the single-factor structure of the scale.

**Table 5:** Goodness of fit indices for the confirmatory factor analysis of the FS

Models	χ2	*df*	Normed χ2	RMSEA	SRMR	CFI	GFI
Model 1	316.22	20	15.8	0.13	0.07	0.85	0.91
Model 2	244.87	19	12.9	0.12	0.06	0.89	0.93
Model 3	189.06	18	10.5	0.11	0.06	0.91	0.95
Model 4	146.27	17	8.6	0.09	0.05	0.93	0.96
Model 5	117.36	16	7.3	0.09	0.04	0.95	0.97
Model 6	77.61	15	5.2	0.07	0.03	0.97	0.98
Model 7	61.15	14	4.4	0.06	0.03	0.98	0.98

Model 1: baseline model; model 2: items 6 and 8’s errors covaried; model 3: items 4 and 8’s errors covaried; model 4: items 6 and 7’s errors covaried; model 5: items 2 and 4’s errors covaried; model 6: items 2 and 8’s errors covaried; model 7: items 7 and 8’s errors covaried. In each subsequent model, the error correlations were added to those already added in the previous models

### Convergent and discriminant validity

Table 2 shows the correlation coefficients between the scales. As expected the FS was positively correlated with the SWLS (0.54, *p* < 0.01), with the HS (0.5, *p* < 0.01) and the LS (0.39, *p* < 0.01); whereas it was negatively correlated with the CES-D 8 (− 0.49, *p* < 0.01).

## Discussion

In this paper, we present the adaptation of the FS to Russian language and culture, which is the first measure able to assess psychological functioning in this country. The current study had two objectives. First, we assessed the psychometric properties of the Russian version of the FS. The results confirm the single-factor structure of the scale, but the factor loadings are lower than those reported in previous studies. Second, we measured the level of psychological functioning in a sample of Siberian older adults. Respondents reported to have a positive perception of their flourishing, even if the flourishing score was lower compared to that reported in previous study on older adults [20].

The Russian version of the FS had an internal consistency comparable to that reported in previous studies [1, 20–36]. The Cronbach’s alpha (0.82), the Cronbach’s alpha values if an item is deleted (ranging from 0.78 to 0.81, see Table 3), and the item-total correlations (ranging from 0.54 to 0.76, see Table 3) showed a good correlation without having redundant items. A principal axis factor analysis and a confirmatory factor analysis revealed a single-factor structure. However, factor loadings (ranging from 0.46 to 0.71) and the variance explained (37%) of the principal axis factor analysis were lower than those reported in previous studies [1, 20, 23, 26, 32–35, 38]. The factor loadings (ranging from 0.39 to 0.80, see Table 3) of the confirmatory factor analysis were also lower than those reported in previous studies [20–28, 31–35, 38]. In the confirmatory factor analysis, the standardized factor loadings of some items (e.g., item 2: “*My social relationships are supportive and rewarding*”, loading = 0.39) were lower than expected. Although our results suggest that the Russian version of the FS is a reliable and valid measure of the construct and that a single factor structure fits the data, the low factor loadings points to the need of further investigations of the psychometric properties of the scale, possibly including different age groups (e.g., young adults). It is possible that for Siberian population a single-factor structure does not perfectly fit the data, or that the translation needs further adaptation. Finally, the FS showed high convergence with other scales. The high positive correlations with SWLS, HS, and LS showed a high convergent validity, whereas the high negative correlation with the CES-D 8 indicated a high discriminant validity of our adaptation of the FS. To sum up, these results indicate that the Russian version of the FS had an acceptable reliability and construct validity with a sample of older adults living in Siberia.

Although each item of the FS has been designed to represent a distinct aspect of psychological functioning [1, 28], previous studies allowed error covariance between items in order to reach acceptable values for the fit indices in the confirmatory factor analysis of the one-factor model [20–22, 27, 33]. Different error correlations were implemented into the models: covariation of the error terms between items 4–5, 4–6, 5–6, 6–7, 6–8 in Hone et al.’s study [20]; 2–3, 2–5, 3–5 in Perera et al.’s study [22]; 6–7 in Tong et al.’s study [27]; 1–2, 1–7, 1–8, 6–7, 6–8, 7–8 in Momtaz et al.’s study [33]. In our study, to improve the goodness of fit of the model, we also allowed errors to correlate. However, although some of the error correlations overlap between studies, no clear pattern emerged. Moreover, none of these modifications, both in our study and in the previous ones, were based on theoretical assumptions. Concerning our study, we might speculate that the covariation of the error terms for the items 2 (“*My social relationships are supportive and rewarding*”), 4 (“*I actively contribute to the happiness and well-being of others*”), and 8 (“*People respect me*”) may be due to the fact that they measure social relationships [1]. However, the reason why some items have correlated errors remains unclear and thus it should be further investigated in future studies.

To our knowledge, this study provides the first measure of psychological functioning among older adults living in Siberia. Consistently with previous studies (e.g., [20, 33]), flourishing score was influenced by the demographic characteristics (see Table 1). The mean flourishing score in our sample was 40.9 (SD = 7.1). Considering that the score ranges from 8 to 56, older adults in our sample reported a moderate positive flourishing level, suggesting that they positively perceived the functioning of the socio-psychological aspects of their lives. However, in the study of Hone and colleagues [20], older adults reported a higher mean flourishing level (see Table 4). The lower mean flourishing score reported by our sample might be due to a variety of factors, such as the effects of the extensive political and economic changes occurred in the Russian society after the breakup of the USSR, and the relatively low income that can be obtained through pensions [5–7]. The results are consistent with the lower life satisfaction as reported by measures of the Cantril ladder [41] and the lower life satisfaction and happiness as reported by Didino and colleagues [7]. In the last decades, Russian older adults have witnessed the intense evolution of their society and this could have affected negatively their well-being. Although our sample is not representative of the Russian older adults, our result is consistent with previous research showing that this transition had a negative impact on the well-being of the population [5]. Huppert and So [18], using a different measure, also reported a lower level of flourishing in the Russian population, compared to other European countries. Future studies should further investigate how psychological flourishing differs in different age groups and in different areas in Siberia. Cross-cultural studies, e.g. comparing Russian older adults with east and west European countries, could also help to shed light on the effects of the evolution of the Russian society on the well-being of older adults.

Some limitations of this study should be taken into account and addressed in future studies. First, although the results indicate that the Russian adaptation of the FS have an acceptable reliability and construct validity, the low values of the factor loadings requires that future studies further investigate and confirm the psychometric properties and the measurement invariance of the Russian version of this scale. Second, we only focused on older adults living in Siberia. In order to being able to generalize the reliability and validity of the Russian adaptation, the factor structure should be assessed in different groups (e.g., adolescent or workers). Moreover, Russia is a multi-ethnic and multicultural society and thus the FS should also be tested in these different segments of the population. Third, objective measures of well-being could be included in future studies and compared with the assessment provided by the FS.

## Conclusion

To date, a Russian instrument to assess flourishing does not exist and thus the adaptation of the FS provides Russian researchers with the first instrument capable to investigate this construct. The scale showed an acceptable construct validity, a good internal consistency, and a good convergent validity with other scales measuring well-being. Although our sample reported a moderate positive level of flourishing, the mean score is lower than those reported in other studies, probably due to the long-term consequences of the extensive political and economic changes occurred in the Russian society after the breakup of the USSR. Reliable instruments to measure psychological flourishing are relevant for both researchers and policymakers. In fact, high levels of psychological flourishing are associated with positive outcomes for both the individual (e.g., better health and higher life expectancy) and the society (e.g., being more productive). Moreover, older adults represent a growing segment of the population and thus it is important to investigate the factors that influence their quality of life. The Russian version of the FS provides an important instrument for Russian researchers and policymakers and will allow future studies to better understand the determinants of successful ageing in this society.

### Additional files

Additional file 1: Template of the R script. (R 4 kb)

Additional file 2: Dataset with the variables analysed in this manuscript. (CSV 60 kb)

## Appendix I

### The Russian version of the Flourishing Scale

7-point Likert scale:

Items:

## Abbreviations

CES-D: Center for Epidemiologic Studies–Depression Scale
CFA: Confirmatory factor analysis
FS: Flourishing Scale
HS: Happiness single item
LS: Life Satisfaction single item
PFA: Principal axis factor analysis
SWLS: Satisfaction With Life Scale
